# Supplementary material for: Machine-learning reprogrammable metasurface imager
Source: Nat Commun. 2019 Mar 6;10:1082. doi: 10.1038/s41467-019-09103-2 (PMC6403242; doi:10.1038/s41467-019-09103-2)
Supplement: Supplementary file 1 — Supplementary Information [file 41467_2019_9103_MOESM1_ESM.docx]

Supplementary Information for

**Machine-Learning Reprogrammable Metasurface Imager**

Lianlin Li^1+*^, Hengxin Ruan^1+^, Che Liu^2+^, Ying Li^3+^, Ya Shuang^1^, Andrea Alù^4,5,6*^, Cheng-Wei Qiu^3*^, and Tie Jun Cui^2*^

^1^ School of Electronic Engineering and Computer Sciences, State Key Laboratory of Advanced Optical Communication Systems and Networks, Peking University, Beijing 100871, China

^2^ State Key Laboratory of Millimeter Waves, Southeast University, Nanjing 210096, China

^3^ Department of Electrical and Computer Engineering, National University of Singapore, 4 Engineering Drive 3, Singapore 117583

^4^ Photonics Initiative, Advanced Science Research Center, City University of New York, 85 St. Nicholas Terrace, New York, NY 10031 USA

^5^ Physics Program, The Graduate Center, City University of New York, 365 Fifth Avenue, New York, NY 10016 USA

^6^ Department of Electrical Engineering, City College of New York, New York, NY 10031 USA

^+^ These authors contribute equally in this work.

^*^ To whom correspondence should be addressed. E-mails: [lianlin.li@pku.edu.cn](mailto:lianlin.li@pku.edu.cn), aalu@gc.cuny.edu, [chengwei.qiu@nus.edu.sg](mailto:chengwei.qiu@nus.edu.sg), [tjcui@seu.edu.cn](mailto:tjcui@seu.edu.cn)

**Supplementary Note 1. Forward Propagation Model of Digital Coding Metasurface**

Here, we apply the technique of induced current inversion^24^ to model the current source $\mathbf{J}^{(S)}$ induced within the macro metasurface particles illuminated by a linearly polarized plane wave, where the superscript $S=00$ (01, 10, 11) corresponds to the particle at the “00” (“01”,”10”, “11”) state. For a metasurface hologram with $N_{x}\times N_{y}$ controllable particles illuminated by the plane wave, the resultant co-polarized radiation in the image plane is approximately written as

$\mathbf{E}\left( \boldsymbol{r} \right)=\sum_{n_{x}=1}^{N_{x}} \sum_{n_{y}=1}^{N_{y}} \int_{\Delta} g(\mathbf{r},\mathbf{r}_{n_{x},n_{y}}+\delta\mathbf{r})\mathbf{J}^{(S_{n_{x},n_{y}})}\boldsymbol{(}\delta\mathbf{r}\boldsymbol{)}d\delta\mathbf{r}$ (1)

where$g\left( \mathbf{r},\mathbf{r}_{n_{x},n_{y}} \right)=\frac{exp\left( -jk_{0}\left| \mathbf{r}-\mathbf{r}_{n_{x},n_{y}} \right| \right)}{4\pi\left| \mathbf{r}-\mathbf{r}_{n_{x},n_{y}} \right|}$ is the three-dimensional free-space Green’s function, $k_{0}$ is the wavenumber, and $\mathbf{r}_{n_{x},n_{y}}$ denotes the central coordinate of the ($n_{x},n_{y}$)th macro metasurface particle. In Eq. (1), the integration is implemented over the surface of the macro metasurface particle denoted by $\Delta$, and the double summation is performed over all pixels of the coding metasurface. $S_{n_{x},n_{y}}$ represents the state of the ($n_{x},n_{y}$)th pixel. Additionally, $n_{x}$ and $n_{y}$ denote the running indices of the pixel of the 2-bit coding metasurface along the $x$ and $y$ directions, respectively.

**Supplementary Note 2. Training Methods**

The machine learning procedure is briefly outlined here. As described in **Methods**, the entries of the measurement matrix are approximately determined by $\mathbf{E}_{i}^{T}(\mathbf{r}_{j})\cdot\mathbf{E}_{i}^{R}(\mathbf{r}_{j})$, where $\mathbf{E}_{i}^{R}\left( \mathbf{r}_{j} \right)$ could be approximately regarded as that of plane wave, since a single receiver is in the far-field region of scene. Then, the entries of measurement matrix can be almost determined by the illumination pattern $\mathbf{E}_{i}^{T}\left( \mathbf{r}_{j} \right)$ of digital metasurface. In this way, the goal of machine learning becomes the design of the desirable radiation patterns of digital metasurface. Taking the PCA training as example, the operational procedure can be summarized as the following three-step method. First, we collect a set of training samples of optical images, and generate the theoretical PCA modes. Second, the modified G-S algorithm is implemented to produce the coding patterns of the digital metasurface in order to generate the desirable radiation patterns whose amplitude distributions are consistent with those of theoretical PCA modes. Finally, in order to calibrate further possible errors of digital metasurface and improve the quality of image reconstruction, the measurement matrix $\mathbf{H}$is updated by solving the linear inverse problem of $\mathbf{Y=HX}$, where P-column matrix $\mathbf{X}$ denotes the collected P training optical images, and $\mathbf{Y}$ are the corresponding measurements. Note that this inverse problem can be solved by employing the standard minimum least square method.

**Supplementary Note 3. MNIST Dataset**

In our numerical study, the probed objects are modeled by exploring MNIST, a dataset of handwriting digits widely used in the area of machine learning. For the electromagnetic (EM) simulations, the objects are set to be perfect conduct in our numerical and experimental tests.

**Supplementary Note 4. Machine-Learning-Driven Imaging over MNIST Dataset**

We test numerically and experimentally our machine-learning imager over the MINIST dataset. In our study, we fabricate 5000 training samples and 5000 test samples. The theoretical PCA bases and the corresponding experimental radiation patterns are shown in **Supplementary Figures 3a** and **b**, respectively, in which the coding patterns according to the radiation patterns in **Supplementary Figure 3b** are presented in **Supplementary Figure 3c**. This set of figures illustrates that the programmable coding metasurface can be well trained by PCA. This means that it is capable of generating the measurement modes needed by PCA, which establishes a solid foundation for the machine-learning-driven high-performance imaging with significantly reduced measurements.

**Supplementary Figures 3d-f** illustrate the recovered images of ten digit-like objects of the machine-learning imager trained with random projection and PCA with varying numbers of measurements. Here, **Supplementary Figure 3d** presents the retrieved images with ideally theoretical data from the random projection (the first row), PCA (the second row). In each method, the numbers of measurements are 10, 50, 100, 200, and 300 from the left to the right. We also evaluate quantitatively the image qualities with respect to the signal-to-noise ratio (SNR) in **Supplementary Figure 3g**. Similarly, the imaging results from the full-wave simulation data are given in **Supplementary Figures 3e**-**h**, while the corresponding experimental results are demonstrated in **Supplementary Figures 3f**-**i**. As for the full-wave simulations, the method of moment, a full-wave solution to the Maxwell equations, is employed. We clearly observe that the quality of image turns better with the increasing measurements, and PCA behave better than the random projection, especially in the low measurement cases. Moreover, when the machine-learning imager is trained by PCA, acceptable results can be achieved with mere 100 measurements, corresponding to a compression rate of 12.8%.

**Supplementary Note 5. Object Recognition over MNIST Dataset**

**Supplementary Figure 4a** reports the average classification rate of the ten digits in three different ways with varying measurements, where the theoretical classification results of PCA operating on original optical input images are provided as well, referred to ideal PCA. The experimental classification results for the ten digits with PCA and random projection are detailed in **Supplementary Figures 4b-c** with 60 measurements, respectively. It is clear to see that digits ‘1’, ‘6’, and ‘0’ are the best recognized, while ‘2’, ’4’, and ‘5’ are the worst recognized. Some high misclassification rates (>10%) could occur. For instances, ‘2’ can be misclassified as ‘1’, and ‘4’ and ‘9’ are misclassified with each other quite often. This set of figures clearly indicates that the machine-learning imager trained by PCA can achieve their own theoretical upper limits of classification, and moreover, they are remarkably higher than that of the random projection. Moreover, when the machine-learning imager is trained by PCA, acceptable classification results can be obtained with around 60 measurements, corresponding to a compression rate of only 7.6%.

**Supplementary Figures**

**
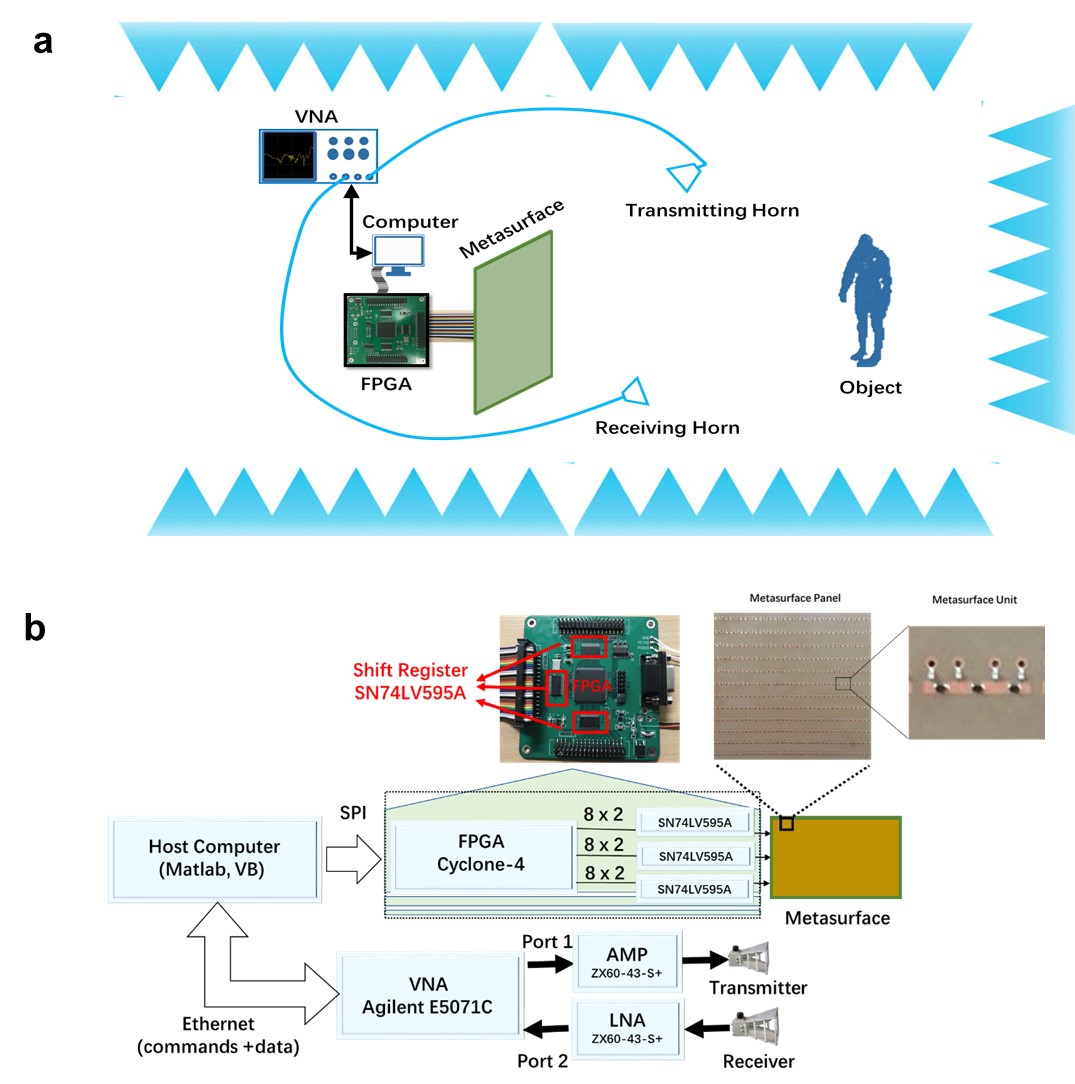
**

**Supplementary Figure 1 | (a) Schematic map of the proposed proof-of-concept system. (b) Schematic block diagram of the proposed imager.**

**
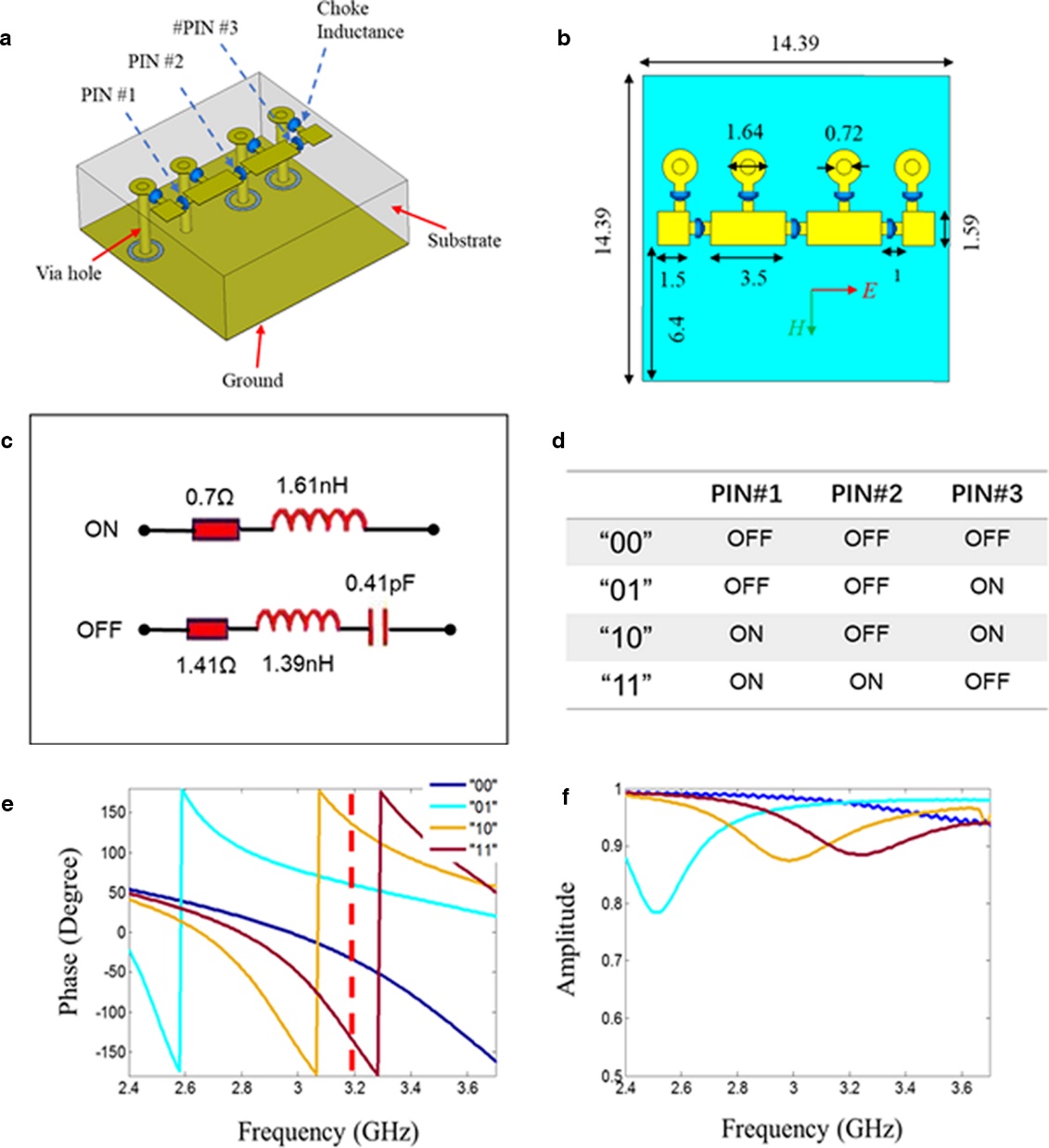
**

**Supplementary Figure 2 | The meta-atom for realizing the 2-bit reprogrammable coding metasurface, the effective circuit models of biased diode, and the corresponding four quantized response statuses.** (**a**) Schematic figure and (**b**) top view of the metamaterial particle to realize the coding metasurface with detailed geometrical parameters. **c**, The effective circuit models of the diode biased at the ‘ON’ and ‘OFF’ states. **d**, The corresponding phase responses of the metamaterial particle as the diode is biased at ‘OFF’ and ‘ON’ state over a range of frequencies. (**e**) Phase and (**f**) amplitude of the metasurface supercell, which show the phase differences of 90° between two neighbored states around 3.0GHz.


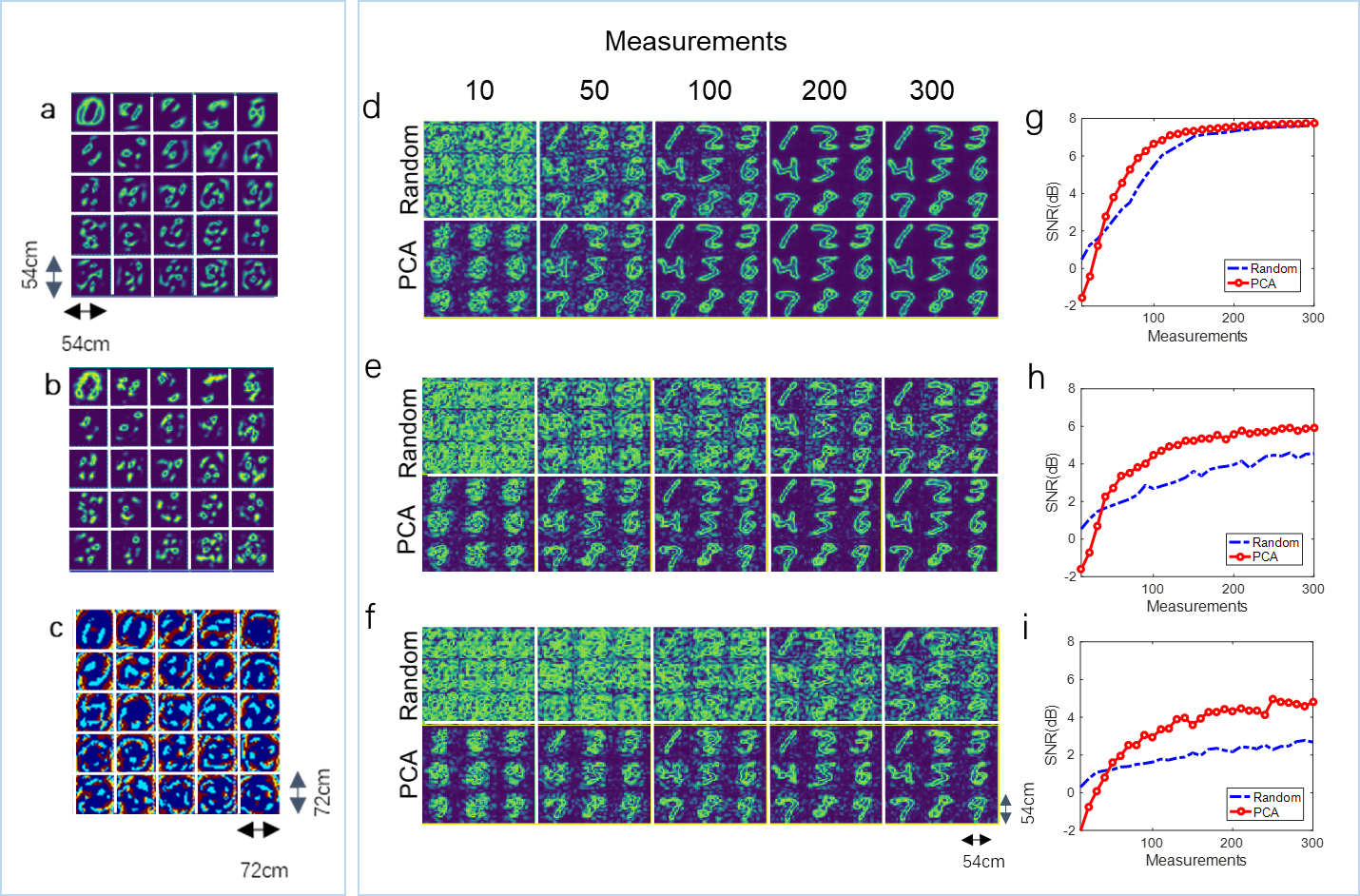


**Supplementary Figure 3 |** **Machine-learning-driven imaging results by the** **real-time digital- metasurface imager trained with random projection and PCA.** **a**, The theoretical PCA bases. **b**, The corresponding experimental radiation patterns. **c**, The coding patterns of metasurface corresponding to the radiation patterns of (**b**). **d**, The theoretical results. **e**, The full-wave simulation results. **f**, The experimental results, in which the first, and second correspond to the random propagation, and PCA, respectively. **g**-**i**, The behaviors of image qualities (for (**g**) theory, (**h**) simulation, and (**i**) experiment results) characterized by SNRs as a function of the number of measurements.


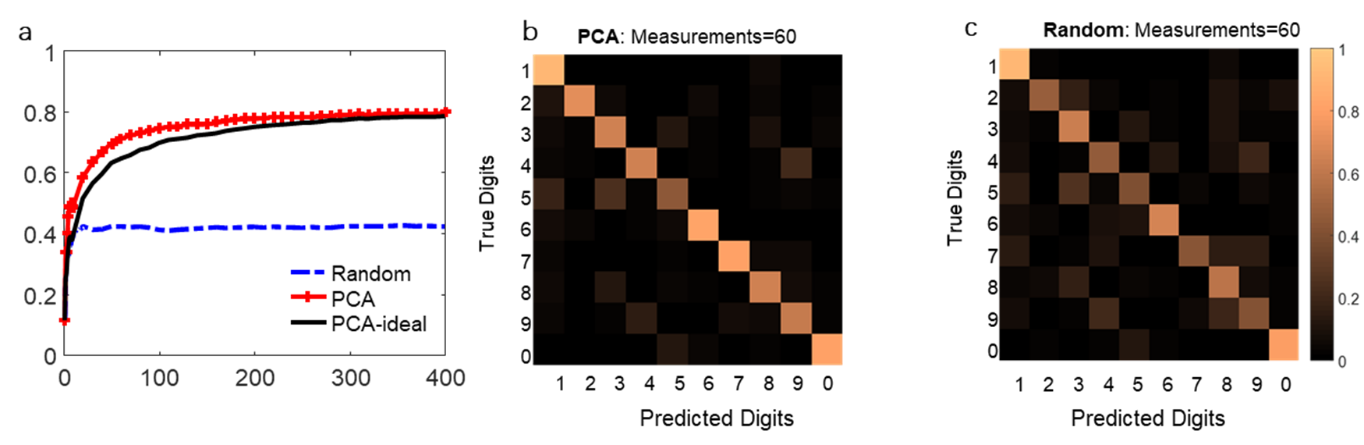


**Supplementary Figure 4 |** **Experimental results of recognition of digit-like objects.** The reprogrammable digital-metasurface imager is trained by three different machine learning techniques of random projection, and PCA. **a**, The classification rates versus the number of measurements. **b-c**, The specific classification results of ten digits in percentage when the imager is trained by the (**b**) PCA, and (**c**) random projection, respectively, where 60 measurements are considered.
