# Supplementary material for: Machine-learning reprogrammable metasurface imager
Source: Nat Commun. 2019 Mar 6;10:1082. doi: 10.1038/s41467-019-09103-2 (PMC6403242; doi:10.1038/s41467-019-09103-2)
Supplement: Supplementary file 9 — Description of Additional Supplementary Files [file 41467_2019_9103_MOESM9_ESM.docx]

**Title: Supplementary Video 1.
Description:** This video records the movement of the training person (Hengxin Ruan, coauthor) without scissors.

**Title: Supplementary Video 2.
Description:** This video records the movement of the training person (Hengxin Ruan, coauthor) with a scissors.

**Title: Supplementary Video 3.
Description:** This video records the reconstructions of the testing person (Ya Shuang, coauthor) without scissors.

**Title: Supplementary Video 4.
Description:** This video records the reconstructions of the testing person (Ya Shuang, coauthor) with scissors.

**Title: Supplementary Video 5.
Description:** This video reports the reconstruction of the testing person (Ya Shuang, coauthor) moving behind an 3cm-thickness opaque wall.

**Title: Supplementary Video 6.
Description:** This video demonstrates the reconstructions of the testing person (Ya Shuang, coauthor) with the growth of the number of measurements.
